# Plasma Fatty Acid Composition, Oxidative and Inflammatory Status, and Adherence to the Mediterranean Diet of Patients with Non-Alcoholic Fatty Liver Disease

**DOI:** 10.3390/antiox12081554

**Published:** 2023-08-03

**Authors:** Margalida Monserrat-Mesquida, Maria Magdalena Quetglas-Llabrés, Cristina Bouzas, Oscar Pastor, Lucía Ugarriza, Isabel Llompart, Karla Cevallos-Ibarra, Antoni Sureda, Josep A. Tur

**Affiliations:** 1 Research Group on Community Nutrition & Oxidative Stress, University of the Balearic Islands-IUNICS, E-07122 Palma de Mallorca, Spain; 2 CIBEROBN (Physiopathology of Obesity and Nutrition), Instituto de Salud Carlos III, E-28029 Madrid, Spain; 3 Health Research Institute of Balearic Islands (IdISBa), E-07120 Palma de Mallorca, Spain; 4 Service of Clinical Biochemistry, Hospital Universitario Ramon y Cajal-IRYCIS, E-28023 Madrid, Spainkarla.cevallos@crc.es (K.C.-I.); 5 C.S. Camp Redó, IBSalut, E-07010 Palma de Mallorca, Spain; 6 Clinical Analysis Service, University Hospital Son Espases, E-07198 Palma de Mallorca, Spain

**Keywords:** hepatic steatosis, Mediterranean diet, fatty acids, inflammation, ultra-processed foods

## Abstract

Non-alcoholic fatty liver disease (NAFLD) is a complex and increasingly prevalent cardiometabolic disorder worldwide. As of today, NAFLD is a pathology without specific pharmacological treatment, with the Mediterranean diet (MedDiet) being the most widely used approach for its management. The objective of this study is to assess the effects of adherence to the Mediterranean diet on fatty acid plasma levels, as well as on the oxidative and inflammatory status of NAFLD patients. A total of 100 adult patients (40–60 years old) diagnosed with NAFLD and from the Balearic Islands, Spain, were classified into three groups according to their adherence to the MedDiet. Consumption was assessed using a validated 143-item semiquantitative Food Frequency Questionnaire. Food items (g/day) were categorised according to their processing using the NOVA system. Anthropometrics, blood pressure, aminotransferases, Dietary Inflammatory Index (DII), inflammatory biomarkers, and fatty acid levels were measured in the plasma of NAFLD patients. High adherence to the MedDiet is associated to a highly plant-based diet, low ultra-processed food (UPF) consumption, low intake of dietary lipids, low intake of animal fats, high intake of monounsaturated fatty acid (MUFA; mainly palmitoleic acid), low intake of saturated fatty acids (SFAs; practically all dietary SFAs), low intake of trans-fatty acids, high intake of omega-3 fatty acids (mainly eicosapentaenoic acid), a higher n-6:n-3 in ratio, low intake of omega-6 fatty acids, and a low level of interleukin-6 (IL-6). High adherence to the MedDiet is related to a better fatty acid profile in the plasma, fewer SFAs and more MUFA and polyunsaturated fatty acids (PUFAs), a plasma biochemical profile, better proinflammatory status, and decreased ultra-processed food consumption of NAFLD patients.

## 1. Introduction

Non-alcoholic fatty liver disease (NAFLD) is a comorbidity of obesity, which is developed after excessive accumulation of triglycerides in the hepatocytes of the liver parenchyma [1]. If this disease is not treated properly, it could progress to non-alcoholic steatohepatitis (NASH), to cirrhosis, and even to hepatocarcinoma [2]. NAFLD is related to metabolic syndrome (MetS), which is associated with central obesity, insulin resistance, hyperglycaemia, hypertension, and dyslipidaemia, considering these the hepatic manifestations of the MetS [3].

Unhealthy diet is an essential factor in the progression of NAFLD [4] since there is strong evidence that the Mediterranean diet (MedDiet) is recommended as a main treatment for the management of NAFLD [5]. Plant-based diets, such as the Dietary Approaches to Stop Hypertension (DASH) and the MedDiet, with recommendations like low intake of saturated fatty acids (SFAs), red and processed meats, and refined carbohydrates (CHOs) are beneficial for NAFLD [6].

Supplementation with omega-3 polyunsaturated fatty acids (PUFAs) could be considered as a therapeutic option in the treatment of NAFLD, because it has been seen that it improves aminotransferase levels—aspartate aminotransferase (AST), alanine aminotransferase (ALT)—and gamma-glutamyl transferase (GGT) [7]. High dietary intake of omega-3 PUFAs, especially those present in the MedDiet could reduce the incidence of major cardiovascular events in patients with MetS [8,9].

The consumption of ultra-processed foods (UPFs), which are industrial formulations manufactured with poor nutritional composition, is harmful to the health, because it induces high levels of NAFLD-related biomarkers and increased age-related accumulation of visceral and total adiposity [10,11].

The gold standard to monitor omega-3 fatty acid input in the organism is the measurement of the total fatty acids in the plasma. Recent studies showed that fatty acid determination could be useful to follow the omega-3 fatty acid input into the plasma in nutritional studies, such as with patients with cystic fibrosis [12] and MetS [9].

Moreover, high adherence to the MedDiet is related to better values of intrahepatic fat content (IFC) in NAFLD patients and a better status of MetS characteristics, such as high decreases in body weight, body mass index (BMI), and blood pressure [13]. A high adherence to the MedDiet is associated with high improvements in the proinflammatory and pro-oxidative status and the cardiorespiratory fitness of NAFLD patients [14].

Therefore, the aim of this study was to assess the effects of adherence to the Mediterranean diet on fatty acid plasma levels, as well as on the oxidative and inflammatory statuses of NAFLD patients.

## 2. Methods

### 2.1. Design and Participants

A total of 100 adults living in Mallorca, Balearic Islands, Spain, were included in the Prevention and Reversion of NAFLD in Obese Patients with Metabolic Syndrome using the Mediterranean Diet and Physical Activity (FLIPAN) trial. The FLIPAN study focused on evaluating the influence of nutritional and physical activity strategies of patients with obesity and NAFLD and, hence, reducing the health burden carried and allowing the early diagnosis of NAFLD. Patients were selected considering the following inclusion criteria: aged 40–60 years old, diagnosis of NAFLD by magnetic resonance imaging (MRI), body mass index (BMI) 27–40 kg/m^2^, and at least three of the five criteria of metabolic syndrome (MetS) according to the International Diabetes Federation (IDF) consensus [15]. The exclusion criteria applied were the same as previously described [16]. Participants were informed of the intention and the repercussions of the trial, and all gave their written consent to participate. The study protocol complied with the Declaration of Helsinki ethical standard, and the experimental procedure was approved by the Balearic Islands Ethics Committee (ref. IB 2251/14 PI). Protocol details are in ClinicalTrials.gov ref. NCT0442620 [17].

The diagnosis of NAFLD was carried out using a 1.5-T magnetic resonance imaging (MRI) machine (Signa Explorer 1.5T, General Electric Healthcare, Chicago, IL, USA) with a 12-channel phased-array coil [18]. The imaging procedure included the IDEAL IQ sequence, which gives, in a non-invasive manner, volumetric whole-liver coverage in a single breath-hold and produces estimated T2* and triglyceride fat fraction maps [19]. Abdominal MRI allows quantification of the liver fat as a mean percentage (IFC), and a mean IFC ≥ 6.4% was established as clinically relevant [20].

### 2.2. Adherence to the Mediterranean Diet

Adherence to the MedDiet was evaluated by using a previously validated questionnaire made up of 17 items [21]. A score was stipulated for each met objective: 0 (non-compliance) or 1 (compliance). The total score ranged between 0 and 17, in which 0 signified no compliance and 17 signified maximum adherence. Participants were distributed into three groups according to the scores obtained in the questionnaire. Therefore, the 1st tertile included patients with low adherence to the MedDiet (<7 points); the 2nd tertile contained participants with moderate adherence to the MedDiet (7–9 points), and finally, the 3rd tertile involved those ones with high adherence to the MedDiet (>9 points).

### 2.3. Anthropometrics and Blood Pressure

Body weight (kg) was measured using a segmental body composition analyser for impedance testing (Tanita MC780P-MA, Tanita, Tokyo, Japan) with participants in bare feet and light clothes, subtracting 0.6 kg. Their body mass indexes (BMIs, kg/m^2^) were then calculated by dividing the weight (kg) by the square of the height (m). Blood pressure was measured with a semi-automatic oscillometer (Omron HEM, 705CP, Hoofddorp, the Netherlands) on the non-dominant arm after 5 min rest in a seated position.

### 2.4. Biochemical Parameters

Blood samples from the antecubital vein were collected in the morning after 12 h overnight fasting conditions, using vacutainers containing ethylenediaminetetraacetic acid (EDTA) as an anticoagulant. Measures of glucose, glycosylated haemoglobin (Hb1ac), triglycerides (TG), high-density lipoprotein-cholesterol (HDL-cholesterol), low-density lipoprotein-cholesterol (LDL-cholesterol), total cholesterol, aspartate aminotransferase (AST), alanine aminotransferase (ALT), gamma-glutamyl transferase (GGT), c-reactive protein (CRP), and total bilirubin were determined in the serum by the Abbot ARCHITECT c16000 using standardised kits (Abbott Diagnostics, Lake Forest, IL, USA). Plasma samples were obtained by centrifuging the blood at 1700× *g* for 15 min at 4 °C.

### 2.5. Dietary Inflammatory Index

The Dietary Inflammatory Index (DII) was calculated to determine the inflammatory potential of a diet using the previously described procedure [22]. This score integrates the effects of 45 food items on 6 inflammatory biomarkers (IL-1b, IL-4, IL-6, IL-10, tumour necrosis factor-alpha (TNFα), and highly sensitive C-reactive protein (CRP)). The DII is based on dietary intake derived from the validated FFQ used in the FLIPAN trial [18].

Foods were assigned values with a negative score if their effect was anti-inflammatory, a positive score if their effect was proinflammatory, and 0 if there was no evidence of a significant change in inflammatory biomarkers [23]. The values of each food component analysed were subtracted from the mean value of the general database for the index and then divided by the standard deviation. The obtained values were translated into percentiles and multiplied by the specific inflammatory score of the global parameter of the food. At last, the total of all the specific DII scores provided the overall DII scores for everyone. Thus, positive DII scores indicated a proinflammatory diet whereas negative DII scores represented a more anti-inflammatory diet [24].

### 2.6. Dietary Intake Assessment

Dietary intakes were obtained using a validated 148-item food frequency questionnaire (FFQ) [25,26]. The items signified the consumption of usual portion sizes of food and drink; moreover, participants were requested at what frequency they consumed an item assessed on the FFQ during the past year. The ratio n-6/n-3 PUFA was calculated by dividing the sum of the linoleic and arachidonic acid by the sum of the linolenic acid, eicosapentaenoic acid (EPA), docosapentaenoic acid (DPA), docosahexaenoic acid (DHA), and omega-3 of non-animal origin. Spanish food composition tables [27] were used to calculate nutrient intakes and to determine consumption of specific food groups. Items of the FFQ were categorised according to the NOVA system [28,29]. The system differentiated four groups: (G1) unprocessed or minimally processed foods, (G2) processed culinary ingredients, (G3) processed foods, and (G4) ultra-processed foods. Details on the distribution of the items of the FFQ into the four NOVA groups are described in Table 1.

### 2.7. Oxidative Stress and Inflammatory Biomarkers

As oxidative stress biomarkers, superoxide dismutase (SOD) and catalase (CAT) enzymatic activity were measured in the plasma. Moreover, as proinflammatory cytokines, interleukin-6 (IL-6) and tumour necrosis factor-alpha (TNFα) were determined in the plasma. Specifically, CAT activity was measured by the spectrophotometric method of Aebi based on the decomposition of H_2_O_2_ [30], whereas SOD activity was analysed using an adaption of the method of McCord and Fridovich [31]. Both enzymatic activities were determined using a spectrophotometer Shimadzu UV-2100 (Shimadzu Corporation, Kyoto, Japan) at 37 °C. TNFα and IL-6 were measured in the plasma using Human Custom ProxartPlex^TM^ (Invitrogen—Thermo Fisher Scientific, Vienna, Austria) following the provided instructions.

### 2.8. Gas Chromatography–Mass Spectrometry (GC/MS) Analysis of Fatty Acids

The fatty acid profile of the plasma was determined using gas chromatography–mass spectrometry (GC/MS). Plasma samples (50 μL) were subjected to transesterification to obtain the fatty acid methyl esters (FAMEs) following the method described by Lepage and Roy [32]. An internal standard (C23:0 methyl ester) was added to all samples to control recovery. The dry plasma lipid extract was dissolved in 150 μL of hexane, and FAMEs were separated and analysed using an Agilent 6890 N GC and an Agilent 5975 MS detector (Agilent Technologies, Santa Clara, CA, USA) with an HP-INNOWAX capillary column (J&W Scientific, Santa Clara, CA, USA) measuring 25 m × 0.20 mm with a 0.20 mm film thickness. Helium was used as the carrier gas at a flow rate of 1.0 mL/min and variable pressure depending on the retention time for arachidonic acid (C20:0). The inlet temperature was controlled at 250 °C. The oven temperature was initially held at 50 °C for 2 min and then increased to 200 °C at 25 °C/min and then to 230 °C at 1.5 °C/min and held at 230 °C for 8 min. The total run time was 35 min. The injector was set to split mode (split ratio 1:50, injection volume 1 μL). The mass spectrometry (MS) transfer line was maintained at a temperature of 260 °C.

The MS parameters were set to an electron ionization of 70 eV. Peak identification was achieved both by comparison with known external standards and by monitoring characteristic ions as described elsewhere [33]. For quantization purposes, the MS detector was operated in selective ion-monitoring mode following at least one quantifier and two qualifying ions for each FAMEs. Concentrations of FAMEs in plasma were obtained from external calibration curves constructed from an external standard mixture (GLC-462, Nu-Chek Prep, Elysian, MN, USA).

### 2.9. Statistics

Statistical analysis was carried out with the Statistical Package for Social Sciences (SPSS v.28 for Windows, IBM Software Group, Chicago, IL, USA). Data were achieved at baseline. Results are represented as the mean with standard deviation (SD), and for all statistics, the level of significance was established at *p* < 0.05. Participants were classified in tertiles according to their adherence to the MedDiet. The normal distribution of the continuous data was assessed using histograms and normal probability plots. The statistical significance of the data was checked with one-way analysis of variance (ANOVA) when variables fit a normal distribution or the Kruskal–Wallis test for non-normally distributed variables. Bonferroni’s post hoc test was performed when significant differences were obtained between groups. Sex, as a categorical variable, was expressed as a percentage and was analysed using the χ^2^ test.

## 3. Results

The results of this study were classified in tertiles according to the adherence to a MedDiet. Tertile 1 was low (<7); tertile 2 was moderate (7–9); and tertile 3 was high (>9) adherence to the MedDiet.

The characteristics of participants classified according to their adherence to the MedDiet are shown in Table 2. Triglycerides and GGT were lower in the participants with high adherence to the MedDiet than the low-adhered group to the MedDiet. Trends of decreases in weight, systolic and diastolic blood pressure, LDL-cholesterol, total cholesterol, aminotransferases (AST and ALT), total bilirubin, and IFC levels were observed in patients with high adherence to the MedDiet. The DII was lower in participants with high adherence to the MedDiet.

Dietary intakes according to adherence to the MedDiet are shown in Table 3. Animal fat intake showed a trend to be lower and plant-based fat to be higher among high-adherence participants than those with low adherence. Monounsaturated fatty acid (MUFA) and PUFA intakes showed a trend to be higher and SFA intake lower in high-adherence participants. EPA, DPA, and DHA were demonstrated with higher intake by participants with high adherence to the MedDiet than by those in tertile 1. Ratio n-6/n-3 PUFAs were higher in participants with low adherence to the MedDiet than those in tertile 3. However, trans-fatty acid intakes showed trends to be lower in participants with high adherence to the MedDiet. The consumption of unprocessed or minimally processed foods was higher and the consumption of UPFs was lower in participants with high adherence to the MedDiet than those in tertile 1.

**Figure 1:**
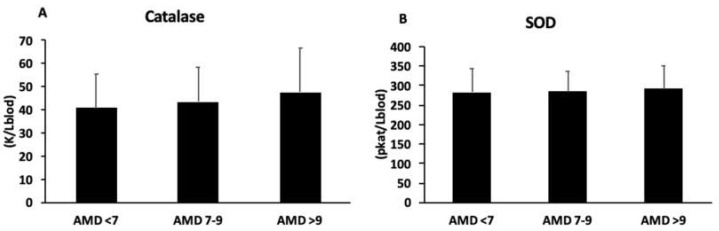
Catalase (**A**) and superoxide dismutase (**B**) activity in plasma, classified according to adherence to MedDiet (AMD). Statistical analysis: one-way ANOVA. Results are presented as mean (SD).

The plasma activity of catalase and superoxide dismutase (SOD) is presented in Figure 1A,B, respectively. No differences were shown in catalase and SOD activity between groups.

**Figure 2:**
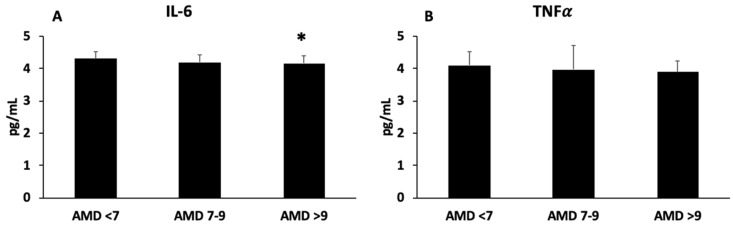
Interleukin-6 (**A**) and tumour necrosis factor alpha (**B**) levels in plasma, classified according to adherence to MedDiet (AMD). Statistical analysis: one-way ANOVA. Results are presented as mean (SD). * Differences with respect to ADM < 7 group when *p* < 0.05.

The plasma levels of IL-6 and TNFα are shown in Figure 2A,B, respectively. The levels of IL-6 were lower in participants with high adherence to the MedDiet than those in the ADM < 7 group, whereas no differences were observed in TNFα levels.

The plasma concentration of fatty acid in participants according to MedDiet adherence is shown in Table 4. SFA levels (myristic acid, palmitic acid, stearic acid, and docosanoic acid) showed a significant decrease in participants with medium and high adherence to the MedDiet with respect to the low-adhered group. Lignoceric acid was significantly reduced in the high-adhered group compared to the lower ones. There were no significant differences in the content of arachidonic acid between groups.

In MUFA concentrations, especially of palmitoleic acid, significant reductions were seen with medium and high adherence to the MedDiet when they were compared to the low-adhered group. No significances were appreciated in the levels of omega-9 fatty acids or oleic acid.

The omega-6 fatty acids determined in this study were linoleic acid, gamma-linoleic acid, dihomo-γ-linolenic acid, arachidonic acid, adrenic acid, and docosapentaenoic acid (DPA n-6). All of them, except for arachidonic acid, showed significantly lower values in the medium- and high-adherence tertiles than the lower-adherence group.

The omega-3 fatty acids measured were alfa-linoleic acid (ALA), EPA, DPA, and DHA. All of them were higher in the group with higher adherence to the MedDiet with respect to ADM < 7, although only the EPA levels were significant.

Determination of the total lipids as fatty acid methyl esters (FAMEs) was performed in the plasma of NAFLD patients; the main result for this determination is that participants in moderate adherence and high adherence presented significantly reduced levels of FAMEs than participants with low adherence to the MedDiet.

## 4. Discussion

The main findings of this study are that intake and plasma levels of fatty acids are clearly related to adherence to the MedDiet and better proinflammatory status. Thus, higher adherence to the MedDiet is associated with a vegetable-rich diet, low UPF consumption, low intake of dietary lipids, low intake of animal fats, high intake of MUFA (mainly palmitoleic acid), low intake of SFAs (practically all dietary SFAs), low intake of trans-fatty acids, high intake of omega-3 fatty acids (mainly EPA), a higher n-6/n-3 in ratio, low intake of omega-6 fatty acids, and a low level of IL-6.

SFAs were lower in participants with the highest adherence to the MedDiet. SFAs are associated with a higher inflammatory state due to their ability to activate the Toll-like receptor (TLR)-2 and -4 pathways, which are innate immune receptors that lead to the activation of NADPH oxidase and the production of proinflammatory cytokines [34]. It was shown that adherence to healthy dietary patterns was related to a decreased risk of MetS, so the increment of SFA intake was associated with higher features of MetS [35]. The results also revealed increased levels of n-6 PUFA in the participants with lower adherence to the MedDiet. It was found that there are diets which have deficiencies in omega-3 fatty acids and have excessive quantities of omega-6 fatty acids, promoting many pathologies, such as cardiovascular disease, cancer, and inflammatory and autoimmune disease [36]. This fact was mainly due to the ability of dietary LA to activate the expression cyclooxygenase-2 (COX-2), promoting the production of proinflammatory eicosanoids from arachidonic acid [37]. Also, a direct association was observed in this study between n-3 PUFA plasma levels and adherence to the MedDiet.

The current results also revealed that participants with better adherence to the MedDiet showed healthier biochemical characteristics. These results are in accordance with previous studies which reported high blood glucose and HbA1c levels in diabetic patients with low adherence to the MedDiet [38,39]. Moreover, a previous study related the consumption of dietary fat to an increased risk of having cardiovascular disease, MetS, and hyperglycaemia [40]. The current results evidenced a triglyceride reduction as well as a slight although not significant decrease in the total cholesterol of participants with high adherence to the MedDiet and confirm that the MedDiet contributes to reducing the incidence of cardiovascular events. It was suggested that the anti-inflammatory properties characteristic of the MedDiet pattern could explain, at least in part, the beneficial effects of high adherence [41].

Better evolution of GGT was observed in the group with higher adherence to the MedDiet. These findings agree with previous studies of NAFLD patients which confirmed that the MedDiet contributes to lowering the plasma levels of liver enzymes and, also, decreases the risk and severity of NAFLD [42,43]. In the current study, an inverse association was observed between the adherence to the MedDiet and the IFC determined with magnetic resonance, and similarly, a significant reduction in IFC was evidenced in NAFLD patients after 6 months of consuming the MedDiet [14]. In agreement with the current results, it was reported that the decrease in IFC was more pronounced in a hypocaloric MedDiet than in a low-fat diet and leading to better improvement of cardiometabolic parameters [44]. The current results confirm the association between better adherence to the MedDiet and increased consumption of MUFA and PUFA fatty acids. It is in accordance with previous studies, which evidenced that the MedDiet is very rich in unsaturated fatty acids and polyphenols, having beneficial effects on cardiovascular disease and NAFLD [45,46]. The reduction in SFAs in patients with more adherence to the MedDiet could be associated with the consumption of more fibre, since it was demonstrated that patients ingesting extra fibre showed a significant increase in MUFA and a decrease in SFAs and, consequently, low fatty liver [47].

Omega 3, especially EPA, was significantly higher in the participants with highest adherence to the MedDiet. Omega-3 PUFAs are fundamental in the human diet, and dietary supplementation with omega-3 PUFAs has been proposed to be effective to manage NAFLD as well as to improve hepatic steatosis [48,49]. The obtained results showed more intake of linoleic acid and a higher n-6/n-3 ratio in the low-adhered group. It has been observed that a low n-6/n-3 PUFA ratio (4:1) leads to a reduction in ALT, triglycerides, LDL-cholesterol, and an improvement in whole-body insulin sensitivity, favouring the reversion of fatty liver disease [50]. It was observed that supplementation with omega-3 PUFAs, especially DHA and EPA, may be effective as a treatment in the early stages of NAFLD [51]. After a one-year omega-3 PUFA treatment for NAFLD patients, a significant reduction in GGT activity, a decrease in liver fat, and beneficial changes in the plasma lipid profile were evidenced [52,53].

The replacement of SFAs by MUFA and PUFAs was linked to a reduction in MetS components, suggesting that emphasizing the qualities of fat intake, rather than just reducing total fat intake, is important for health benefits [54]. The current results revealed lower levels of trans-fatty acids and UPFs in the group with higher adherence to the MedDiet. It was shown that a higher consumption of UPFs was directly correlated with adiposity accumulation in an elderly population with chronic health conditions [11]. A prospective study of older adults with chronic health conditions revealed that consumption of UPFs was positively associated with the fatty liver index and hepatic steatosis index [10], which are usually used as indirect measures of NAFLD [55,56].

In contrast, the current study reveals that those patients with higher adherence to the Mediterranean diet showed a higher intake of fruits and vegetables than those with lower adherence. An intake of foods that provide antioxidants, especially fruits, vegetables, and extra virgin olive oil, like in the Mediterranean diet, can increase the antioxidant response in the body. The Mediterranean diet shows beneficial effects due to the antioxidant and anti-inflammatory properties of most of its components, such as polyphenols like resveratrol, MUFA and PUFA, tocopherol, and flavonoids, which showed positive effects related to hypertension, obesity, and hypercholesterolemia in the prevention of cardiovascular disease [57].

The increase in n-3 PUFA in the plasma of participants with the highest adherence to the MedDiet suggests that the MedDiet could be a good treatment against NAFLD. There is evidence that plasma n-3 fatty acids are inversely associated with macrovascular disease and death by type 2 diabetes, supporting the cardioprotective effects of n-3 PUFAs [58]. Moreover, it was shown that omega-3 PUFAs, such as EPA and DHA, generate pro-resolving mediators like lipoxins, resolvins, protectins, and maresins. These pro-resolving mediators are involved in the process of inflammation resolution [59]. Thus, a better diet with appropriate levels of n-3 PUFAs is related to a better anti-inflammatory profile. In addition, n-3 PUFAs interact with peroxisome proliferator-activated receptor-α (PPAR-α), causing its activation and the upregulation of the hepatic energy-sensing cascade that increases fatty acid oxidation and reduces de novo fatty acid synthesis, contributing to reducing liver steatosis [60]. However, a low synthesis of n-3 PUFAs was described in patients with NAFLD, which was associated with a decrease in the activity of desaturase enzymes and even an increase in oxidative stress [61]. A previous study showed lower liver delta-5 and delta-6 desaturase (D-5D and D.6D) activity in obese NAFLD patients than in non-obese patients [62]. In addition, a study using a mouse model evidenced that the supplementation of hydroxytyrosol, which is a bioactive compound with antioxidant activity typical of the MedDiet, was capable of normalizing desaturase activity and reducing fat accumulation in the liver [63].

The DII classifies individual diets according to their inflammatory potential. Current results reported significantly lower values of the DII in participants with higher adherence to the MedDiet. The lower values of the DII and IFC showed that a diet with a better inflammatory profile could be associated with a decreased risk of NAFLD severity. According to this statement, proinflammatory diets were related with a high risk of severe NAFLD independently of potential confounders such as MetS components [64].

The current patients with better adherence to the MedDiet showed higher levels of antioxidant enzymatic activity in their plasma, as well as better proinflammatory status than those ones with lower adherence to the MedDiet. These results are in accordance with a previous study evidencing high levels of enzymatic activity of catalase and SOD in plasma from patients with high cardiovascular risk after an intervention with the MedDiet [65]. Moreover, it was observed that after 6 months of lifestyle intervention, IL-6 and TNFα were decreased, although only significantly for IL-6 in the group with high adherence to the MedDiet [14]. Some of the beneficial effects of the MedDiet are related to the presence of bioactive compounds with antioxidant and anti-inflammatory activity [66,67]. Specifically, bioactive ingredients such as polyphenols, MUFAs and PUFAs, and fibre together could increase the antioxidant potential and decrease the inflammatory state, while also decreasing LDL and cholesterol synthesis and improving insulin sensitivity and endothelial function [68].

## 5. Strengths and Limitations

The main strength of this study is that an association has been found between plasma fatty acids, oxidative stress, and proinflammatory biomarkers and adherence to the MedDiet. However, this study also shows several limitations. The sample size was relatively small; however, the sample size was enough to evidence the existence of differences in the biomarker levels and plasma fatty acid composition between levels of adherence to the MedDiet stages by NAFLD patients. More parameters on oxidative stress or antioxidant response in plasma and erythrocytes could be assessed.

## 6. Conclusions

The current study showed that higher adherence to the MedDiet is related to a better plasma biochemical profile, as well as a better proinflammatory status in NAFLD patients. This higher adherence implies a better quality of the diet that is evidenced by a lower value of the DII, a more balanced consumption of fatty acids with a decreased n-6/n-3 ratio, and low UPF consumption. Fatty acids in the plasma of participants also showed a better FA profile in plasma with a low intake of SFAs and a high intake of MUFAs and PUFAs, especially EPA, which can contribute to an anti-inflammatory status and a reduction in steatosis. In the absence of a specific pharmacological treatment for NALFD, the current results show the importance of the diet to better manage and reverse the hepatic steatosis.

## Figures and Tables

**Table 1:** Distribution of items of the FFQ into four groups according to the degree of processing established by NOVA classification system.

Group 1: Unprocessed or minimally processed foods	Milk (whole-fat, semi-skimmed, and skimmed), yogurt (whole-fat and skimmed), eggs, meats (chicken, turkey, beef, pork, lamb, rabbit), liver, offal, fish and seafoods, fresh vegetables, gazpacho, boiled potatoes, fresh fruits, dried fruits, nuts, legumes, whole-grain cereals, rice (whole-grain and refined), pasta (whole-grain and refined), natural fruit juices, coffee, and tea.
Group 2: Processed culinary ingredients	Vegetable oils (regular and virgin extra olive oil, oils from sunflower seeds, corn, and soybeans), butter, lard, salt, sugar, and honey.
Group 3: Processed foods	Condensed milk, cream, cheeses (cured, semi-cured, cottage, and fresh), bacon, cured ham, canned fish, home-made French fries, olives, fruits in syrup, breads (white and whole grain), marmalade, decaffeinated coffee, beer, wine, and champagne.
Group 4: Ultra-processed foods	Milkshakes, petit-suisse, custard, ice cream, processed meat (ham, chorizo, mortadella, sausages, hamburgers, meat balls, pate), potato chips, breakfast cereals, cookies, industrial and commercial pastries (croissants, donuts, muffins, cakes, churros), chocolates, sugary cocoa powder, marzipan, nougat, creamy cheese spreads, margarine, pre-prepared dishes (croquettes, pizza), instant soups, mayonnaise, mustard, ketchup, packed fried tomato sauce, savoury packed snacks, commercial fruit juices, alcoholic drinks produced by fermentation followed by distillation (liquors, whisky, vodka, gin, liquors).

**Table 2:** Characteristics of participants stratified by adherence to Mediterranean diet.

	AMD < 7 n = 30	AMD 7–9 n = 35	AMD > 9 n = 35	*p*-Value
Sex (%)				
Men	56.5	56.0	45.5	0.782
Women	43.5	44.0	54.5	
	**Mean (SD)**	**Mean (SD)**	**Mean (SD)**	
Age (years)	50.1 (6.8)	51.8 (7.8)	54.8 (5.9) *	0.007
Weight (kg)	93.9 (12.3)	92.3 (10.4)	90.3 (8.6)	0.473
BMI (kg/m^2^)	32.7 (2.8)	32.5 (3.9)	33.4 (2.8)	0.336
Systolic blood pressure (mmHg)	136.8 (13.4)	135.9 (10.6)	135.3 (14.1)	0.857
Diastolic blood pressure (mmHg)	82.4 (8.0)	82.9 (7.0)	81.6 (6.3)	0.652
Glucose (mg/dL)	117.2 (63.1)	105.5 (21.8)	110.5 (32.6)	0.475
HBA1c (%)	6.08 (1.69)	5.80 (0.81)	6.01 (1.19)	0.572
Triglycerides (mg/dL)	217.1 (121.4)	158.1 (77.7) *	151.4 (69.3) *	0.006
HDL-cholesterol (mg/dL)	42.1 (7.7)	45.9 (13.6)	45.7 (8.2)	0.255
LDL-cholesterol (mg/dL)	135.1 (26.3)	133.5 (36.4)	127.6 (31.9)	0.588
Total cholesterol (mg/dL)	223.7 (41.4)	217.4 (59.7)	203.4 (35.3)	0.190
AST (U/L)	24.5 (10.0)	24.6 (12.8)	22.9 (7.07)	0.775
ALT (U/L)	31.1 (17.4)	27.7 (11.5)	25.7 (10.5)	0.284
GGT (U/L)	42.4 (23.6)	31.9 (14.3) *	33.3 (14.1) *	0.048
CRP (mg/dL)	0.570 (0.841)	0.489 (0.554)	0.468 (0.458)	0.776
Bilirubin total (mg/dL)	0.713 (0.411)	0.717 (0.294)	0.689 (0.346)	0.934
IFC (%)	13.1 (9.0)	12.8 (9.9)	12.0 (8.59)	0.869
DII	0.751 (2.1)	0.559 (2.5)	−0.693 (2.1) *#	0.011

Abbreviations: AMD, adherence to Mediterranean diet; BMI, body mass index; Hb1Ac, glycated haemoglobin 1A; HDL-cholesterol, high-density lipoprotein; LDL-cholesterol, low-density lipoprotein; AST, aspartate aminotransferase; ALT, alanine aminotransferase; GGT, gamma glutamyl transferase; CRP, c-reactive protein; DII, Dietary Inflammatory Index; SD, standard deviation. Results from categorical variables are expressed as percentages and analysed by χ^2^, whereas quantitative variables are expressed as mean (SD). Statistics achieved by one-way ANOVA or Kruskal–Wallis. * Differences vs. ADM < 7. # Differences vs. ADM 7–9. Data points are significant when *p* < 0.05.

**Table 3:** Dietary intakes of antioxidants and fatty acids of patients with NAFLD stratified by adherence to Mediterranean diet.

	AMD < 7 n = 30	AMD 7–9 n = 35	AMD > 9 n = 35	*p*-Value
	Mean (SD)	Mean (SD)	Mean (SD)	
Fruits (g/day)	217.3 (199.3)	299.4 (199.7)	372.5 (213.6) *	0.032
Vegetables (g/day)	284.9 (187.8)	321.3 (193.8)	351.9 (190.2)	0.302
Extra virgin olive oil (g/day)	14.4 (16.1)	17.7 (3.4)	17.4 (3.5)	0.079
Fat from foods of animal origin (g/day)	49.1 (24.3)	45.7 (16.1)	42.7 (18.5)	0.544
Plant-based fat (g/day)	54.2 (21.4)	57.0 (18.5)	63.3 (33.1)	0.471
MUFA (g/day)	48.7 (21.5)	47.3 (13.8)	50.9 (20.6)	0.786
PUFAs (g/day)	17.5 (7.7)	16.2 (5.3)	18.3 (11.7)	0.693
SFAs (g/day)	27.2 (12.1)	26.4 (7.6)	26.2 (10.7)	0.938
Cholesterol (mg/day)	445.4 (178.6)	378.8 (115.8)	375.7 (134.6)	0.205
Phytosterols (mg/day)	331.7 (95.9)	325.4 (91.3)	382.9 (136.3)	0.139
Linoleic acid (g/day)	12.5 (6.0)	11.1 (3.9)	11.5 (5.4)	0.679
Linolenic acid (g/day)	1.17 (0.54)	1.18 (0.42)	1.35 (0.77)	0.505
Arachidonic acid (g/day)	0.130 (0.059)	0.151 (0.067)	0.138 (0.065)	0.423
Omega-3 EPA + DPA + DHA (g/day)	0.575 (0.331)	0.599 (0.326)	0.976 (0.651) *#	0.009
Omega-3 from fish + EPA+ DHA (g/day)	0.372 (0.165)	0.570 (0.393)	0.746 (0.511) *	0.016
Omega-3 non-animal origin (g/day)	0.116 (0.060)	0.093 (0.045)	0.082 (0.045)	0.083
Ratio n-6:n-3	5.98 (1.87)	4.88 (1.52)	4.17 (1.82) *	0.013
Trans-fatty acids (g/day)	0.729 (0.451)	0.577 (0.205)	0.564 (0.295)	0.189
Unprocessed or minimally processed foods (% of consumption)	59.8 (16.9)	67.6 (13.9)	72.5 (11.5) *	0.013
Processed culinary ingredients (% of consumption)	2.1 (1.0)	2.2 (1.3)	2.6 (1.8)	0.471
Processed foods (% of consumption)	15.9 (9.4)	15.8 (8.7)	14.4 (5.3)	0.776
Ultra-processed food (% of consumption)	33.3 (20.7)	14.6 (13.3) *	12.7 (16.5) *	<0.001

Abbreviations: AMD, adherence to Mediterranean diet; MUFA, monounsaturated fatty acid; PUFA, polyunsaturated fatty acid; SFAs, saturated fatty acids; EPA, eicosapentaenoic acid; DPA, docosapentaenoic acid; DHA, docosahexaenoic acid; SD, standard deviation. Results are expressed as mean (SD). Statistics achieved by one-way ANOVA or Kruskal–Wallis. * Difference with respect to ADM < 7. # Difference with respect to ADM 7–9. Data points are significant when *p* < 0.05.

**Table 4:** Fatty acids in the plasma of participants stratified by adherence to Mediterranean diet.

	AMD < 7 n = 30	AMD 7–9 n = 35	AMD > 9 n = 35	*p*-Value
	Mean (SD)	Mean (SD)	Mean (SD)	
SFAs				
Myristic acid, C14:0 (nM)	110.1 (34.8)	77.3 (21.9) *	79.4 (21.7) *	<0.001
Palmitic acid, C16:0 (nM)	1651.3 (331.8)	1410.0 (215.8) *	1477.3 (278.2) *	<0.001
Stearic acid, C18:0 (nM)	640.3 (73.7)	592.6 (65.7) *	585.1 (64.3) *	0.005
Arachidic acid, C20:0 (nM)	13.9 (1.9)	13.4 (1.3)	13.8 (1.5)	0.413
Docosanoic acid, C22:0 (nM)	30.2 (5.1)	27.1 (4.3) *	27.1 (4.9) *	0.014
Lignoceric acid, C24:0 (nM)	31.1(7.5)	27.1 (7.0)	26.0 (5.9) *	0.010
MUFA				
Palmitoleic acid, C16:1 (nM)	120.3 (45.5)	95.6 (43.4) *	83.6 (26.9) *	0.002
n-9 PUFA				
Oleic acid, C18:1 n9 cis/trans (nM)	1228.6 (445.1)	1060.8 (304.4)	1088.7 (348.4)	0.133
n-6 PUFAs				
Linoleic acid, C18:2 n6, LA (nM)	1724.9 (326.5)	1352.3 (272.3) *	1389.3 (211.2) *	<0.001
Gamma-linoleic acid, C18:3 n6 (nM)	35.7 (13.0)	26.0 (8.3) *	24.3 (10.5) *	<0.001
Dihomo-y-linoleic acid C20:3 n6 (nM)	118.1 (24.8)	99.6 (27.0) *	96.3 (27.0) *	0.004
Arachidonic acid, C20:4 n6 (nM)	464.9 (129.1)	443.5 (122.2)	414.6 (119.8)	0.265
Adrenic acid, C22:4 n6 (nM)	21.0 (7.8)	15.1 (5.0) *	13.9 (5.1) *	<0.001
Docosapentaenoic acid (DPA), C22:5 n6 (nM)	16.4 (6.6)	14.4 (6.1) *	11.3 (4.1) *	0.007
n-3 PUFAs				
Alfa-linoleic acid (ALA), C18:3 n3 (nM)	19.8 (6.0)	18.4 (6.4)	19.7 (6.1)	0.568
Eicosapentaenoic acid (EPA), C20:5 n3 (nM)	25.0 (12.1)	32.8 (14.2)	37.0 (17.5) *	0.008
Docosapentaenoic acid (DPA), C22:5 n3 (nM)	25.8 (7.1)	25.3 (6.2)	26.2 (7.0)	0.833
Docosahexaeonic acid (DHA), C22:6 n3 (nM)	118.1 (32.2)	123.4 (34.7)	127.7 (27.0)	0.495
FAME Total (nM)	6521.6 (1090.1)	5757.1 (966.0) *	5749.6 (1021.9) *	0.003

Abbreviations: AMD, adherence to Mediterranean diet; MUFA, monounsaturated fatty acid; PUFA, polyunsaturated fatty acid; SFAs, saturated fatty acids; n-9, omega-9 fatty acids; n-6, omega-6 fatty acids; n-3, omega-3 fatty acids; FAMEs, fatty acid methyl esters; SD, standard deviation. Results are expressed as mean (SD). Statistics achieved by one-way ANOVA or Kruskal–Wallis. * Difference with respect to ADM < 7. Data points are significant when *p* < 0.05.

## Data Availability

There are restrictions on the availability of data for this trial due to the signed consent agreements around data sharing, which only allow access to external researchers for studies following the current project’s purposes. Researchers wishing to access the trial data used in this study can make a request to the corresponding author: pep.tur@uib.es.
